# 

**Published:** 1860-07

**Authors:** 


### DENTAL REGISTER

### OF THE WEST.

EDITED BY

J. TAFT AND GEO. WATT.

July, 1860.

MONTHLY.--THREE DOLLARS PER ANNUM IN ADVANCE.

CINCINNATI:
J. T. TOLAND, PUBLISHER AND PROPRIETOR.

